# Intranasal administration of a single dose of MVA-based vaccine candidates against COVID-19 induced local and systemic immune responses and protects mice from a lethal SARS-CoV-2 infection

**DOI:** 10.3389/fimmu.2022.995235

**Published:** 2022-09-12

**Authors:** Patricia Pérez, David Astorgano, Guillermo Albericio, Sara Flores, Pedro J. Sánchez-Cordón, Joanna Luczkowiak, Rafael Delgado, José M. Casasnovas, Mariano Esteban, Juan García-Arriaza

**Affiliations:** ^1^ Department of Molecular and Cellular Biology, Centro Nacional de Biotecnología (CNB), Consejo Superior de Investigaciones Científicas (CSIC), Madrid, Spain; ^2^ Centro de Investigación Biomédica en Red de Enfermedades Infecciosas (CIBERINFEC), Madrid, Spain; ^3^ Pathology Department, Centro de Investigación en Sanidad Animal (CISA), Instituto Nacional de Investigación y Tecnología Agraria y Alimentaria (INIA), Consejo Superior de Investigaciones Científicas (CSIC), Madrid, Spain; ^4^ Department of Microbiology, Instituto de Investigación Hospital Universitario 12 de Octubre (imas12), Madrid, Spain; ^5^ Department of Medicine, School of Medicine, Universidad Complutense de Madrid, Madrid, Spain; ^6^ Department of Macromolecular Structures, Centro Nacional de Biotecnología (CNB), Consejo Superior de Investigaciones Científicas (CSIC), Madrid, Spain

**Keywords:** SARS-CoV-2, vaccine candidates, MVA, S protein, intranasal delivery, immunogenicity, protective efficacy, mice

## Abstract

Current coronavirus disease-19 (COVID-19) vaccines are administered by the intramuscular route, but this vaccine administration failed to prevent severe acute respiratory syndrome coronavirus 2 (SARS-CoV-2) virus infection in the upper respiratory tract, mainly due to the absence of virus-specific mucosal immune responses. It is hypothesized that intranasal (IN) vaccination could induce both mucosal and systemic immune responses that blocked SARS-CoV-2 transmission and COVID-19 progression. Here, we evaluated in mice IN administration of three modified vaccinia virus Ankara (MVA)-based vaccine candidates expressing the SARS-CoV-2 spike (S) protein, either the full-length native S or a prefusion-stabilized [S(3P)] protein; SARS-CoV-2-specific immune responses and efficacy were determined after a single IN vaccine application. Results showed that in C57BL/6 mice, MVA-based vaccine candidates elicited S-specific IgG and IgA antibodies in serum and bronchoalveolar lavages, respectively, and neutralizing antibodies against parental and SARS-CoV-2 variants of concern (VoC), with MVA-S(3P) being the most immunogenic vaccine candidate. IN vaccine administration also induced polyfunctional S-specific Th1-skewed CD4^+^ and cytotoxic CD8^+^ T-cell immune responses locally (in lungs and bronchoalveolar lymph nodes) or systemically (in spleen). Remarkably, a single IN vaccine dose protected susceptible K18-hACE2 transgenic mice from morbidity and mortality caused by SARS-CoV-2 infection, with MVA-S(3P) being the most effective candidate. Infectious SARS-CoV-2 viruses were undetectable in lungs and nasal washes, correlating with high titers of S-specific IgGs and neutralizing antibodies against parental SARS-CoV-2 and several VoC. Moreover, low histopathological lung lesions and low levels of pro-inflammatory cytokines in lungs and nasal washes were detected in vaccinated animals. These results demonstrated that a single IN inoculation of our MVA-based vaccine candidates induced potent immune responses, either locally or systemically, and protected animal models from COVID-19. These results also identified an effective vaccine administration route to induce mucosal immunity that should prevent SARS-CoV-2 host-to-host transmission.

## Introduction

Severe acute respiratory syndrome coronavirus 2 (SARS-CoV-2), the causal agent of the coronavirus disease-19 (COVID-19) pandemic (1, 2), has infected more than 559 million people worldwide since December 2019, and has caused around 6.3 million deaths. The rapid development and administration of several vaccines among the human population helped to control the disease, contributing to diminish the incidence of the virus, reducing hospitalizations and mortality. However, the pandemic is still ongoing in spite of vaccination with millions of infections and deaths, mostly due to the appearance of SARS-CoV-2 variants of concern (VoC) with increased transmissibility, as current vaccines do not prevent respiratory infections. Thus, novel immunization regimens and optimized vaccines able to induce mucosal and systemic long-term immune responses that could control respiratory viral infections and protect against different VoC are needed to stop the pandemic.

Most of the preclinical and clinical trials against COVID-19 have used vaccines administered by the intramuscular route. However, intramuscular immunization failed to prevent SARS-CoV-2 virus infection in the upper respiratory tract, mainly due to the absence of virus-specific mucosal immune responses, as the first line of protection against airway infection comes from mucosal membranes (3). The intranasal (IN) immunization route is known to induce mucosal immunity, activating the innate immune system and eliciting antigen-specific humoral and cellular immune responses, all contributing to the clearance of the virus (4–6). Accordingly, IN vaccine administration has been tested against SARS-CoV-2 infection, confirming that this immunization route reinforces local immune responses and is highly effective (3, 7–11). Therefore, in principle, a mucosal immunization, such as the IN route, could be a suitable COVID-19 vaccination procedure able to trigger both systemic and mucosal immune responses, leading to better control of SARS-CoV-2 replication and transmission than the delivery of vaccines by the intramuscular route.

Poxvirus vectors, which include vaccinia virus (VACV) and its highly attenuated strain modified vaccinia virus Ankara (MVA), are the most intensively studied of the Orthopoxvirus genus; poxvirus-based vaccine candidates expressing heterologous antigens have been used with promising results in numerous animal models and in diverse clinical trials against several pathogens (12, 13). Moreover, MVA has been approved as a smallpox vaccine in the United States, Canada, and the European Union. Recently, with the appearance of the COVID-19 pandemic, we and others have generated several MVA vectors expressing SARS-CoV-2 antigens that induce, upon intramuscular immunization, robust immune responses, both humoral and cellular, leading to high efficacy against SARS-CoV-2 in several animal models (mice, hamsters, and macaques) (14–28). Moreover, a recent phase 1 clinical trial showed safety and immunogenicity of a synthetic MVA vector expressing the S and N proteins of SARS-CoV-2 (29). In particular, we generated COVID-19 vaccine candidates based on the MVA vector expressing either a human codon optimized full-length native spike (S) protein (MVA-CoV2-S and MVA-Δ-CoV2-S) or a full-length S protein stabilized in the prefusion conformation [MVA-CoV2-S(3P)]. Intramuscular administration of one or two doses of these vaccine candidates induced potent SARS-CoV-2-specific T-cell and humoral immune responses in mice, hamsters, or rhesus macaques, as well as full protection against SARS-CoV-2 infection in those animal models (16, 19, 20, 22, 25, 26).

Although systemic inoculation was the most frequent route of poxvirus vaccination, the use of poxvirus vectors as mucosal vaccines has been documented (30). In fact, MVA administration by a mucosal route was first described in 1972, whereupon following IN inoculation, MVA conferred protection against a poxvirus challenge in monkeys and rodents (31). Later, several studies reported that MVA-based vaccines administered intranasally were effective to induce a protective response against respiratory viruses, such as influenza (32), parainfluenza (33), or respiratory syncytial virus (34) in different models, confirming that IN administration of MVA-based vaccines induced a highly efficient local and systemic immune response against several pathogens. Recently, several studies started to explore the IN administration route to improve the action of MVA-based vaccine candidates against COVID-19, with promising results (14, 15).

Here, we have evaluated the immunogenicity and efficacy in mice of three MVA-based vaccine candidates against COVID-19, expressing either a native non-stabilized or a prefusion-stabilized SARS-CoV-2 S protein, when administered as a single dose by the IN route. Results showed that all vaccine candidates induced systemic anti-S IgG and mucosal IgA antibodies, neutralizing antibodies against several SARS-CoV-2 VoC, and S-specific local and systemic T-cell immune responses, and fully protected the animals against SARS-CoV-2 infection. MVA-CoV2-S(3P), which express a prefusion-stabilized S protein, was the more immunogenic and efficacious vaccine candidate, eliciting higher titers of S-specific IgG and IgA antibodies and of neutralizing antibodies, and significantly reducing SARS-CoV-2 replication in upper and lower respiratory tracts, lung pathology, and levels of pro-inflammatory cytokines. These results favor IN administration of MVA-based vaccine candidates to prevent COVID-19 and SARS-CoV-2 transmission in humans.

## Materials and methods

### Animals and ethics statement

Female C57BL/6OlaHsd mice (6–8 weeks old) used for immunogenicity assays were purchased from Envigo Laboratories and stored in the animal facility of the Centro Nacional de Biotecnología (CNB) (Madrid, Spain). Female transgenic K18-hACE2 mice, expressing the human angiotensin converting enzyme-2 (ACE2) receptor, were obtained from the Jackson Laboratory [034860-B6.Cg-Tg(K18-ACE2)2Prlmn/J, genetic background C57BL/6J × SJL/J)F2], and efficacy experiments were performed in the biosafety level 3 (BSL-3) facilities at the Centro de Investigación en Sanidad Animal (CISA)-Instituto Nacional de Investigaciones Agrarias (INIA-CSIC) (Valdeolmos, Madrid, Spain). The immunogenicity and efficacy animal studies were approved by the Ethical Committee of Animal Experimentation (CEEA) of the CNB-CSIC and by the Division of Animal Protection of the Comunidad de Madrid (PROEX 49/20, 169.4/20 and 161.5/20). Animal procedures conformed with international guidelines and with Spanish law under the Royal Decree (RD 53/2013).

### MVA-based vaccine candidates

We used the following MVA-based vaccine candidates against COVID-19, whose generation was previously described: (i) MVA-CoV2-S (also termed MVA-S; expressing a human codon optimized full-length non-stabilized SARS-CoV-2 S protein from the Wuhan strain) (20), (ii) MVA-Δ-CoV2-S (also termed MVA-Δ-S; expressing a human codon optimized full-length non-stabilized SARS-CoV-2 S protein from the Wuhan strain, but from an MVA vector containing deletions in the vaccinia virus immunomodulatory genes *C6L*, *K7R*, and *A46R*) (20), and (iii) MVA-CoV2-S(3P) [also termed MVA-S(3P), expressing a human codon optimized full-length prefusion-stabilized SARS-CoV-2 S protein from the Wuhan strain containing three amino acid mutations in the furin cleavage site (R682G, R683S, and R685S) to avoid the cleavage of the S protein in S1 and S2, and three S2 amino acid substitutions to proline to stabilize the S protein in a prefusion conformation (A942P, K986P, and V987P) and to enhance expression of the S protein] (26). Moreover, we used the attenuated MVA-WT poxvirus strain as a control, obtained from the Chorioallantois vaccinia virus Ankara strain after 586 serial passages in chicken embryo fibroblast cells (35). All MVA viruses were grown in cultured chicken cells (DF-1), purified by centrifugation through two 36% (wt/vol) sucrose cushions in 10 mM Tris-HCl (pH 9) and tittered by immunostaining, as previously described (36).

### Immunogenicity study schedule in C57BL/6 mice

To evaluate the immunogenicity of the MVA-based vaccine candidates against COVID-19 after IN administration, groups of female C57BL/6 mice (*n* = 6 per group; 6 to 8 weeks old) were slightly anesthetized with isoflurane (1-chloro-2,2,2-trifluoroethyl difluoromethyl ether; Isoflo^®^, Zoetis Belgium SA) and each mouse received one dose of 1 × 10^7^ plaque-forming units (PFUs) of MVA-S, MVA-Δ-S, or MVA-S(3P) by the IN route in 50 μl of PBS. Mice inoculated with nonrecombinant MVA-WT were used as a control group. No adverse effects were detected in immunized mice. Then, 14 days after the immunization, mice were euthanized by using a lethal dose of 10% xylazine (Xilagesic 20 mg/ml; Laboratorios Calier, Barcelona, Spain) + 10% ketamine (Imalgene 100 mg/ml; Merial Laboratorios, Barcelona, Spain). Blood from each mouse was collected by cardiac puncture, maintained at 37°C for 1 h, kept at 4°C overnight, and centrifuged at 3,600 rpm for 20 min at 4°C to obtain serum samples that were stored at −20°C until used, to analyze SARS-CoV-2-specific humoral immune responses. Bronchoalveolar lavages (BAL) from each mouse were taken by flushing 700 μl of Roswell Park Memorial Institute (RPMI) 1640 medium (Gibco-Life Technologies, Carlsbad, CA, USA) supplemented with HEPES pH 7.4 (10 mM, Merck, Darmstadt, Germany), β-mercaptoethanol (10 µM, Sigma-Aldrich, St. Louis, MO, USA), and L-glutamine (2 mM, Merck, Darmstadt, Germany) into the trachea; then, samples were spun down to remove cellular debris and supernatants were kept at −20°C until used. Spleens, lungs, and bronchoalveolar lymph nodes (BLNs) extracted from each mouse were pooled per group, processed mechanically (spleens and BLNs) or enzyme-digested (lungs), blood-cell depleted, and filtered through 40-µm cell strainers until single-cell samples were obtained that were used to measure the SARS-CoV-2 S-specific T-cell immune responses by an intracellular cytokine staining (ICS) assay.

### ICS assay

The magnitude and polyfunctionality of SARS-CoV-2 S-specific CD4^+^ and CD8^+^ T cells expressing CD107a, and/or IFNγ, and/or TNFα, and/or IL-2 were analyzed by an ICS assay as previously described (20) in cells (splenocytes, lung cells, or bronchial lymph nodes) stimulated with a SARS-CoV-2 S peptide pool (1 µg/ml) (JPT Peptide Technologies, Berlin, Germany), spanning the S1 and S2 regions of the S protein from the Wuhan strain, and containing 158 (S1) and 157 peptides (S2) as consecutive 15-mers overlapping by 11 amino acids. Moreover, the magnitude and polyfunctionality of SARS-CoV-2 S-specific CD4^+^ T follicular helper (Tfh) cells expressing CD154, and/or IFNγ, and/or IL-21 were analyzed by an ICS assay as previously described (20) in splenocytes stimulated with a SARS-CoV-2 S protein (5 µg/ml) plus S1 and S2 peptide pools (1 µg/ml). Cells were acquired with a Gallios flow cytometer (Beckman Coulter), and analyses of the data were performed with the FlowJo software version 10.4.2 (Tree Star), as previously described (20).

### Enzyme-linked immunosorbent assay

The titers of binding anti-S IgG, IgG1, IgG2c, and IgG3 antibodies in individual or pooled sera from immunized C57BL/6 or K18-hACE2 mice were measured by ELISA as previously described (20, 22, 26). Moreover, titers of binding anti-S IgA antibodies were also analyzed in pooled BAL samples. The SARS-CoV-2 S protein used to coat the plates derived from the Wuhan strain (GenBank accession number MN908947.3) was previously described (20, 22, 26). The S protein sequence spans residues 1 to 1,208 and contained a T4 fibritin trimerization sequence, a Flag epitope, and an 8×His-tag at the C-terminus; the furin-recognition motif (RRAR) was replaced by the GSAS sequence, and it also contained the A942P, K986P, and V987P substitutions in the S2 portion. The S protein was purified by nickel-nitrilotriacetic acid (Ni-NTA) affinity chromatography from transfected cell supernatants, and it was transferred to HEPES buffered saline (HBS), pH 7.5, during concentration or by size-exclusion chromatography (SEC). Total binding anti-S IgG and IgA titers were measured as the last serum or BAL dilution, respectively, which gives an absorbance value at 450 nm at least three times higher the absorbance of a naive serum or BAL sample.

### SARS-CoV-2 live neutralization

The capacity of the sera obtained from C57BL/6 or K18-hACE2 immunized mice to neutralize live SARS-CoV-2 virus was measured using a microneutralization test (MNT) assay in a BSL-3 laboratory at the CNB-CSIC, as previously described (16, 22, 25, 26). Serially twofold diluted serum samples in DMEM-2% fetal bovine serum (FBS) medium were incubated at a 1:1 ratio with 100 median tissue culture infectious dose 50 (TCID_50_) of the SARS-CoV-2 MAD6 isolate (similar to the Wuhan strain but having the D614G mutation in the S protein) (37) in 96-well tissue culture plates for 1 h at 37°C. Then, mixtures of serum samples and SARS-CoV-2 were added in triplicate to Vero-E6 cell monolayers seeded in 96-well plates at 2 × 10^4^ cells/well, and plates were incubated at 37°C, in a 5% CO_2_ incubator for 3 days. Then, cells were fixed with 10% formaldehyde for 1 h and stained with crystal violet. When plates were dried, crystal violet was diluted in H_2_O-1% SDS and optical density was measured in a luminometer at 570 nm. Fifty percent neutralization titer (NT_50_) was calculated as the reciprocal dilution resulting in 50% inhibition of cell death following a methodology previously described (38). A WHO International Standard containing pooled plasma obtained from 11 individuals recovered from SARS-CoV-2 infection (NIBSC code: 20/136) was used for the calibration and harmonization of the serological assay detecting anti-SARS-CoV-2 neutralizing antibodies.

### Neutralization of SARS-CoV-2 variants of concern

The capacity of serum samples obtained from C57BL/6 or K18-hACE2 immunized mice to neutralize different SARS-CoV-2 VoC was tested by using SARS-CoV-2 pseudotyped vesicular stomatitis virus (VSV) expressing SARS-CoV-2 S protein, as previously described (22, 25, 26). SARS-CoV-2 S variants used were S_614G, alpha (B.1.1.7), beta (B.1.351), gamma (P.1), delta (B.1.617.2), and omicron (BA.2 and B.1.1.529.2), and were produced as described elsewhere (39). SARS-CoV-2 S mutant D614G was generated by site-directed mutagenesis (Q5 Site Directed Mutagenesis Kit; New England Biolabs) following the manufacturer’s instructions and using as an input DNA a pcDNA3.1 expression vector encoding SARS-CoV-2 S_614D (20). SARS-CoV-2 VoC alpha (B.1.1.7; GISAID: EPI_ISL_608430), beta (B.1.351; GISAID: EPI_ISL_712096), gamma (P.1; GISAID: EPI_ISL_833140), delta (B.1.617.2; GISAID: EPI_ISL_1970335), and omicron (BA.2, B.1.1.529.2; GISAID: EPI_ISL_6640917) were optimized, synthesized, and cloned into pcDNA3.1 by GeneArt (Thermo Fisher Scientific, GeneArt GmbH, Regensburg, Germany). The neutralization activity of serum samples was tested by triplicates at several twofold dilutions. For neutralization experiments, virus-containing transfection supernatants were normalized for infectivity to a multiplicity of infection of 0.5–1 PFU/cell and incubated with the dilutions of serum samples at 37°C for 1 h in 96-well plates. After the incubation time, 2 × 10^4^ Vero-E6 cells were seeded onto the virus–serum mixture and incubated at 37°C for 24 h. Cells were then lysed and assayed for luciferase expression; NT_50_ titers of neutralizing antibodies were determined as the highest serum dilution, which resulted in a 50% reduction of luciferase units compared with pseudotyped viruses not incubated with serum.

### Efficacy study schedule in K18-hACE2 transgenic mice

Female K18-hACE2 mice (9 weeks old at the beginning of the study) immunized with one IN dose of MVA-S, MVA-Δ-S, or MVA-S(3P) were used to evaluate the efficacy of these vaccine candidates. Groups of animals (*n* = 9) received one dose of 1 × 10^7^ PFU of MVA-S, MVA-Δ-S, or MVA-S(3P) by the IN route in 50 μl of PBS. Mice inoculated with nonrecombinant MVA-WT were used as the control group. At week 5 postimmunization, all mice were challenged with a lethal dose (1 × 10^5^ PFU) of SARS-CoV-2 (MAD6 strain) by the IN route in 50 μl of PBS, after being anesthetized in an isoflurane chamber. Mice were then monitored for body weight change and mortality for 14 days postchallenge. Animals with more than 20% weight loss were euthanized by cervical dislocation. At 5 days postchallenge, four mice per group were euthanized, and lung, nasal washes, and serum samples were collected. The entire left lung lobe was removed from each mouse and immersion-fixed in zinc formalin (Sigma-Aldrich) for 48 h. After the fixation period, samples were routinely processed and embedded in paraffin for subsequent histopathological evaluations. Right lung lobes were divided longitudinally into two, with one part placed in RNALater stabilization reagent (Sigma-Aldrich) and stored at −80°C until RNA extraction, and the other lung part was weighted and stored at −80°C until analysis of virus yields. Nasal washes were collected in 100 μl of PBS and stored at −80°C until viral load detection and RNA extraction. Blood was collected by submandibular bleeding, maintained at 37°C for 1 h, kept at 4°C overnight, and centrifuged at 3,600 rpm for 20 min at 4°C to obtain the serum samples, which was then inactivated at 56°C for 30 min and kept at −20°C until use.

### Quantification of SARS-CoV-2 mRNA and cytokine mRNA by reverse transcription-quantitative polymerase chain reaction

Lungs and nasal washes from K18-hACE2 mice were harvested at 5 days postchallenge and were analyzed to quantify SARS-CoV-2 mRNA and cytokine mRNA expression levels using RT-qPCR, as previously described (22, 26). Lungs were stored in RNALater (Sigma-Aldrich) at −80°C until homogenized with a gentleMACS dissociator (Miltenyi Biotec) in 2 ml of RLT buffer (Qiagen) plus β-mercaptoethanol (Sigma-Aldrich). Then, 600 μl of homogenized lung tissue was used to isolate total RNA using the RNeasy Mini Kit (Qiagen), according to the manufacturer’s specifications. On the other hand, 50 μl of nasal washes in PBS was stored at −80°C until RNA extraction using an in-house TRIzol^®^ (Invitrogen) method, as described elsewhere (40). First-strand cDNA synthesis and subsequent real-time PCR were performed using NZYSpeedy One-step RT-qPCR Master Mix (NZYTech), according to the manufacturer’s specifications using ROX as reference dye. SARS-CoV-2 viral mRNA content was determined using a previously validated set of primers and probes specific for the SARS-CoV-2 subgenomic RNA for protein E and the genomic virus RNA-dependent RNA polymerase (RdRp) gene, and specific gene expression was normalized to the expression of the cellular 28S ribosomal RNA gene (41). The mRNA expression levels of several cytokine/chemokine genes (IL-6, IL-12b, CCL2, CCL11, CCL12, IFN-γ, TNF-α, and CXCL10) were analyzed using specific Taqman probes (Thermo Fisher Scientific; sequence will be provided upon request), and their specific gene expression was also presented relative to the expression of the cellular 28S ribosomal RNA gene. Data were acquired with the QuantStudio 5 Real-Time PCR System (Applied Biosystems) and analyzed with QuantStudio Design and Analysis Software v1.5.1 (Applied Biosystems). mRNA arbitrary units (A.U.) were quantified relative to negative RNA samples (from uninfected mice) and were performed using the 2^−ΔΔCt^ method. All samples were tested in duplicate.

### Analysis of SARS-CoV-2 virus yields by plaque assay

Lungs and nasal washes from K18-hACE2 mice were harvested at 5 days postchallenge and were analyzed for the presence of SARS-CoV-2 infectious virus using a plaque assay, as previously described (22, 26). Lungs were harvested, weighted, and stored directly at −80°C until homogenized with a gentleMACS dissociator (Miltenyi Biotec) in 2 ml of PBS buffer. Then, undiluted and serial 10-fold dilutions of homogenized lung tissue or nasal wash samples were added in triplicate to Vero-E6 cell monolayers seeded in 12-well plates at 5 × 10^5^ cells/well, and after 1 h of adsorption, the inoculum was removed and plates were incubated at 37°C, 5% CO_2_ in 2:1 DMEM 2X-4% FBS : Avicel^®^ RC-591 (microcrystalline cellulose and carboxymethylcellulose sodium, DuPont Nutrition Biosciences ApS). After 4 days, cells were fixed for 1 h with 10% formaldehyde (Sigma-Aldrich), and then the supernatant was removed and plaques were visualized by adding 0.5% crystal violet (Sigma-Aldrich). SARS-CoV-2 titers were determined in PFUs per gram of lung tissue or in PFUs per milliliter of nasal wash.

### Lung histopathology

Lung histopathology was performed as previously described (22, 26). The entire left lung lobe was removed from each K18-hACE2 mouse and immersion-fixed in zinc formalin (Sigma-Aldrich) for 48 h. After the fixation period, samples were routinely processed and embedded in paraffin blocks that were then sectioned at 4 µm thickness on a microtome, mounted onto glass slides, and routinely stained with hematoxylin and eosin (H&E). Lung sections were microscopically evaluated using an Olympus BX43 microscope by a single veterinary pathologist who was blinded to the identity and group of individual mice. To assess the character and severity of histopathological lesions, lung inflammation scoring parameters based on previous reports on SARS-CoV-2 infection in mouse models were used (42). The histopathological parameters evaluated were as follows: capillary endothelial cell activation; alveolar hemorrhages; alveolar edema; perivascular edema; alveolar septal thickening (interstitial pneumonia); alveolar damage and hyaline membranes in alveoli; inflammatory cell infiltration in alveoli; bronchi/bronchioles with epithelial necrosis, detached epithelium, or inflammatory cells in the lumen (bronchitis/bronchiolitis); peribronchial/peribronchiolar and perivascular mononuclear infiltrates; pneumocytes hyperplasia; cytopathic effect or syncytia; squamous metaplasia; uniform interstitial fibrosis; organized fibrotic tissue around the bronchi/bronchioles or intrabronchiolar (bronchiolitis obliterans); and pleural thickening. The histopathological parameters were graded following a semi-quantitative scoring system as follows (0): no lesion, (1) minimal lesion, (2) mild lesion, (3) moderate lesion, and (4) severe lesion. The cumulative scores of histopathological lesions provided the total score per animal. In each experimental group, the individual scores were used to calculate the group average. In addition, H&E-stained sections were visually scored 0–6 based on the percentage of lung area affected by inflammatory lesions as follows: 0% of the lung injured (score 0), <5% (score 1), 6%–10% (score 2), 11%–20% (score 3), 21%–30% (score 4), 31%–40% (score 5), and >40% (score 6). In each experimental group, the individual scores were used to calculate the group average.

### Statistical procedures

All the graphs, calculations, and statistical analyses were performed using GraphPad Prism software version 8.0 (GraphPad Software, San Diego, CA, USA). One-way ANOVA of transform data followed by *post-hoc* Student’s *t*-test comparisons was used to establish the differences between two groups. The statistical study of the lung histopathological scores was an unpaired *t*-test. Statistical analysis of the ICS assay data was realized as previously described (43), using an approach that corrects measurements for background response, calculating confidence intervals and *p*-values. Statistical significance is indicated as follows: *p < 0.05; **p < 0.005; ***p < 0.001.

## Results

### One single intranasal dose of MVA-based vaccine candidates in C57BL/6 mice induced systemic S-specific IgGs and neutralizing antibodies against SARS-CoV-2 variants of concern and mucosal S-specific IgA antibodies

To evaluate the SARS-CoV-2-specific humoral immunogenicity induced after IN administration of MVA-based vaccine candidates against COVID-19, C57BL/6 mice (*n* = 6/group) were immunized intranasally with one single dose of 1 × 10^7^ PFUs of MVA-S, MVA-Δ-S, MVA-S(3P), or MVA-WT as a negative control group, and 14 days postimmunization, animals were euthanized and SARS-CoV-2-specific humoral immune responses were evaluated systemically (in serum) or in the mucosa (BAL samples). We used the three MVA recombinant vectors previously generated (20, 22, 26) for comparison of their immunogenicity and efficacy after IN administration.

**Figure 1:**
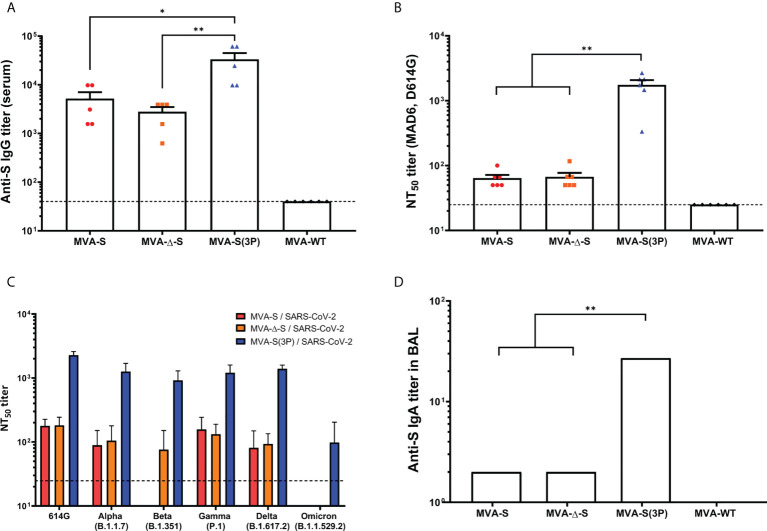
SARS-CoV-2-specific humoral immune responses elicited in C57BL/6 mice immunized with one IN dose of different MVA-based vaccine candidates against COVID-19. SARS-CoV-2-specific humoral immune responses were evaluated in serum and BAL samples obtained at 14 days postimmunization from C57BL/6 mice immunized with one IN dose of MVA-S, MVA-Δ-S, or MVA-S(3P). **(A)** Titers of binding IgG antibodies specific for the S protein (Wuhan strain), determined by ELISA in individual mouse serum samples in duplicate. Mean values and SEM are represented. The dashed line represents the limit of detection. **(B)** SARS-CoV-2 neutralizing antibody titers. NT_50_ titers were determined in individual mouse serum samples by using a live virus microneutralization assay. Mean NT_50_ values and SEM are represented. The dashed line represents the limit of detection (1:25 dilution). **(C)** SARS-CoV-2 neutralizing antibody titers against SARS-CoV-2 VoC. NT_50_ titers were evaluated in pooled mouse serum samples, using VSV-based pseudoparticles expressing the SARS-CoV-2 S protein of different VoC. Mean NT_50_ values and 95% confidence intervals are represented. The dashed line represents the limit of detection. **(D)** Titers of IgA antibodies specific for the S protein (Wuhan strain), determined by ELISA in pooled BAL samples in duplicate. Mean values and SEM are represented. Student’s *t*-test: *p < 0.05; **p < 0.005.

All vaccinated mice induced high titers of anti-S total IgG antibodies in serum samples, but the MVA-S(3P) induced significantly higher titers than MVA-S or MVA-Δ-S (**Figure 1A**). Moreover, all groups of vaccinated mice elicited S-specific IgG1, IgG2c, and IgG3 antibody subclasses with titers of IgG2c > IgG1 > IgG3 and IgG2c/IgG1 ratios above 1 (**Table 1**), indicative of a Th1-like protective immune response.

**Table 1:** Isotype analysis of anti-S IgG antibodies in immunized C57BL/6 and transgenic K18-hACE2 mice.

Time points analyzed	Anti-S IgG1, IgG2c and IgG3 antibody titers and IgG2c/IgG1 ratio in C57BL/6 or transgenic K18-hACE2 vaccinated mice ^a^											
	MVA-S				MVA-Δ-S				MVA-S(3P)			
	IgG1	IgG2c	IgG3	IgG2c /IgG1	IgG1	IgG2c	IgG3	IgG2c /IgG1	IgG1	IgG2c	IgG3	IgG2c /IgG1
Day 14 post-immunization (C57BL/6 mice)	1563	1563	100	1	625	1563	100	2.5	3906	24414	625	6.25
Day 14 post-immunization (prechallenge) (K18-hACE2 mice)	3906	3906	250	1	3906	9766	100	2.5	9766	152588	1563	15.625
Day 5 postchallenge (K18-hACE2 mice)	3906	9766	100	2.5	3906	24414	250	6.25	24414	152588	1563	6.25
Day 14 postchallenge (K18-hACE2 mice)	152588	953674	3906	6.25	61035	953674	9766	15.625	152588	953674	3906	6.25

a Mean titer of IgG1, IgG2c and IgG3 antibody subclasses against SARS-CoV-2 S protein, and IgG2c/IgG1 ratio, from duplicates of pooled sera samples obtained from the different immunization regimens studied.

Next, by using a live microneutralization assay, we detected SARS-CoV-2 neutralizing antibodies against the live parental Wuhan strain virus (MAD6 isolate, containing D614G mutation) in serum samples from all vaccinated mice; a single IN dose of MVA-S(3P) elicited significantly higher neutralizing antibody titers (NT_50_) than MVA-S or MVA-Δ-S, whereas NT_50_ titers corresponding to the control group MVA-WT were background (below the limit of detection; 1:25 dilution) (**Figure 1B**). Furthermore, the analysis in serum samples of neutralizing antibodies against several VoC, by using a VSV-pseudotyped neutralization assay, showed that mice immunized with MVA-S(3P) induced neutralizing antibodies against all VoC tested, with NT_50_ neutralizing antibody titers markedly higher than the titers in MVA-S- or MVA-Δ-S-vaccinated mice (**Figure 1C**). NT_50_ neutralizing antibody titers against SARS-CoV-2 parental D614G mutant, VoC alpha (B.1.1.7), beta (B.1.351), gamma (P.1) and delta (B.1.167.2) were similar, whereas the NT_50_ titers against VoC omicron (BA.2; B.1.1.529.2) were about 10 times lower than for the rest of the variants (**Figure 1C**).

Finally, we evaluated the capacity of the MVA-based vaccine candidates administered intranasally to elicit local mucosal humoral immune responses against SARS-CoV-2, by ELISA analysis of S-specific binding IgA antibodies in BAL samples. The results showed that all vaccinated mice induced anti-S IgA antibodies, with MVA-S(3P) eliciting about 10 times higher titers than MVA-S or MVA-Δ-S (**Figure 1D**).

### MVA-based vaccine candidates administered intranasally in C57BL/6 mice induced local and systemic SARS-CoV-2-specific CD4^+^ and CD8^+^ T-cell immune responses

Next, we evaluated the SARS-CoV-2-specific T-cell immunogenicity induced in C57BL/6 mice (*n* = 6/group) after one single IN dose of 1 × 10^7^ PFUs of MVA-S, MVA-Δ-S, MVA-S(3P), or MVA-WT as a negative control group. At 14 days postimmunization, mice were euthanized and SARS-CoV-2 S-specific T-cell immune responses were evaluated locally (in lungs or bronchial lymph nodes) or systemically (in spleen). Cells were stimulated *ex vivo* with S peptide pools, spanning the entire S protein, and an ICS assay was performed to measure the induction of SARS-CoV-2 S-specific CD4^+^ and CD8^+^ T cells expressing CD107a, and secreting IFN-γ, TNF-α, and/or IL-2.

**Figure 2:**
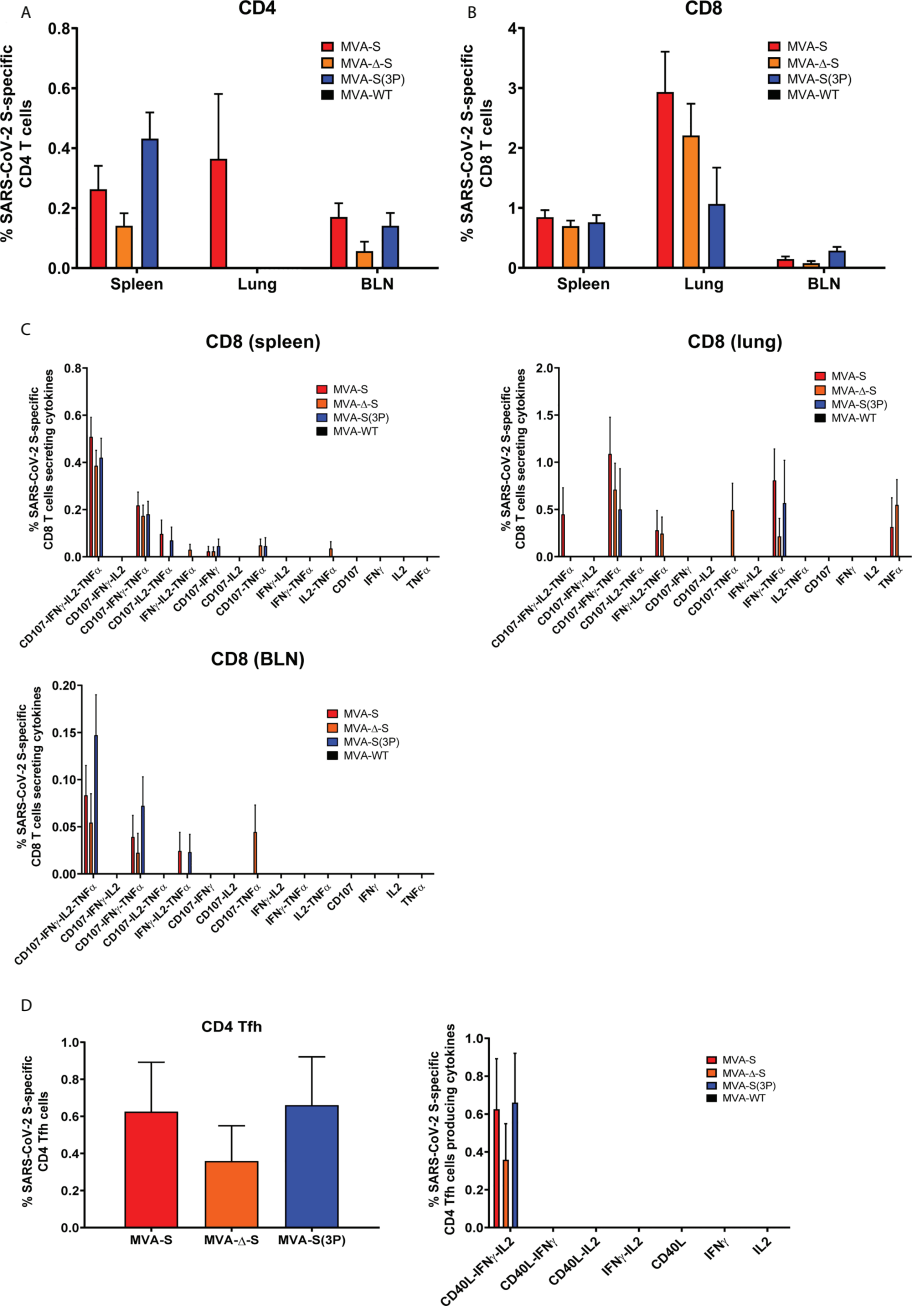
SARS-CoV-2-specific T-cellular immune responses elicited in C57BL/6 mice immunized with one IN dose of different MVA-based vaccine candidates against COVID-19. SARS-CoV-2 S-specific T-cellular immune responses were evaluated in spleens, lungs, and bronchial lymph nodes (BLNs) at 14 days postimmunization from C57BL/6 mice immunized with one IN dose of MVA-S, MVA-Δ-S, or MVA-S(3P). Cell percentages were determined by ICS. **(A, B)** Magnitude of S-specific CD4^+^ **(A)** and CD8^+^ **(B)** T-cell immune responses in spleens, lungs, and BLN. Percentages of CD4^+^ or CD8^+^ T cells expressing CD107a and/or producing IFN-γ and/or TNF-α and/or IL-2 against a mixture of S1 and S2 peptide pools in immunized mice. **(C)** Polyfunctional profiles (based on expression of selected markers CD107a, IFN-γ, TNF-α, and IL-2) of total S-specific CD8^+^ T-cell immune responses directed against a mixture of S1 and S2 peptide pools, in spleen, lungs, and BLN. **(D)** Magnitude of S-specific CD4^+^ Tfh cell responses in spleen. Percentages of CD4^+^ Tfh cells expressing CD40L and/or producing IFN-γ and/or IL-21 against a mixture of S protein plus S1 and S2 peptide pools in immunized mice. Polyfunctionality profile is shown on the right.

All groups of vaccinated mice elicited similar S-specific CD4^+^ and CD8^+^ T-cell immune responses, with a CD4^+^ Th1-skewed profile (**Figure 2A**), and a higher overall response mainly mediated by CD8^+^ T cells of a higher magnitude in the lungs than in spleen (**Figure 2B**). Remarkably, all groups of vaccinated mice triggered highly polyfunctional S-specific CD4^+^ and CD8^+^ T cells, secreting mainly three or four cytokines, either locally (in lungs or bronchial lymph nodes) or systemically (in spleen) (**Figure 2C**).

Moreover, all vaccinated mice induced S-specific CD4^+^ T follicular helper (Tfh) cells expressing CD40L, and/or secreting IFN-γ, and/or IL-21, which were of a similar magnitude and highly polyfunctional with 100% of S-specific CD4^+^ Tfh cells expressing CD40L-IFN-γ-IL-21 (**Figure 2D**).

### Intranasal administration of one single dose of MVA-based vaccine candidates prevented morbidity and mortality of K18-hACE2 transgenic mice challenged with SARS-CoV-2, and reduced SARS-CoV-2 virus replication *in vivo*

**Figure 3:**
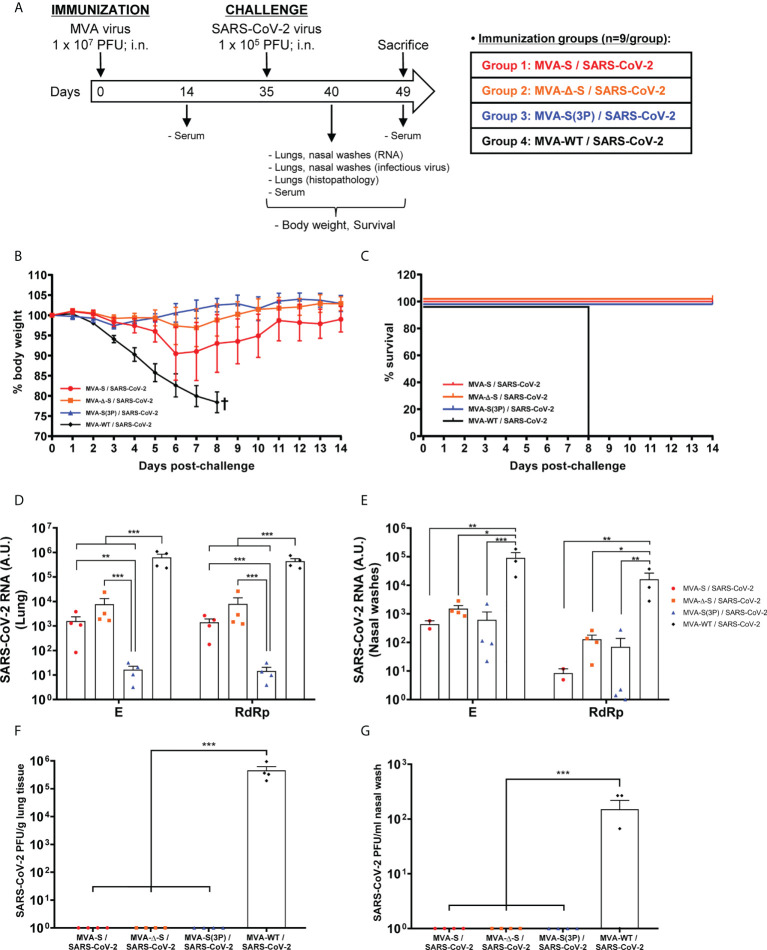
One IN dose of MVA-based vaccine candidates protects transgenic K18-hACE2 mice from SARS-CoV-2 infection. **(A)** Efficacy schedule. Female K18-hACE2 transgenic mice (*n* = 9 per group) were immunized by the IN route with one dose of 1 × 10^7^ PFUs of MVA-S, MVA-Δ-S, or MVA-S(3P) as indicated. At week 5 (day 35), mice were challenged intranasally with 1 × 10^5^ PFUs of SARS-CoV-2 (MAD6 isolate). MVA-WT-inoculated control mice were also challenged with SARS-CoV-2. At day 5 postchallenge, four mice per group were sacrificed and lungs, nasal washes, and serum samples were collected as indicated. Serum was also collected at 14 days after immunization in all groups and at 14 days postchallenge in groups 1, 2, and 3. **(B, C)** The challenged mice were monitored for change of body weight **(B)** and mortality **(C)** for 14 days. †: mice were euthanized due to loss of more than 20% of initial body weight. **(D, E)** Virus replication in lung samples **(D)** and nasal washes **(E)**. SARS-CoV-2 subgenomic E and genomic RdRp mRNA detected by RT-qPCR at 5 days after virus infection (*n* = 4/group). Mean RNA levels (in arbitrary units [A.U.]) and SEM from duplicates of each lung and nasal washes samples; relative values are referred to uninfected mice. **(F, G)** SARS-CoV-2 infectious virus in lung samples **(F)** and nasal washes **(G)**. Mean (PFUs/g of lung tissue or PFUs /ml of nasal wash) and SEM from triplicates of each sample. Student’s *t*-test: *p < 0.05; **p < 0.005; ***p < 0.001.

After the demonstration that one single IN dose of MVA-based vaccine candidates against COVID-19 induced robust humoral and T-cellular immune responses against SARS-CoV-2 in C57BL/6 mice, we next evaluated the efficacy triggered by IN administration of one dose of those vaccine candidates in transgenic K18-hACE2 mice, susceptible to SARS-CoV-2 infection (44, 45). Thus, K18-hACE2 mice (*n* = 9/group) were immunized with one IN dose of 1 × 10^7^ PFUs/mouse of MVA-S, MVA-Δ-S, or MVA-S(3P), and challenged 5 weeks later with a lethal IN dose of 1 × 10^5^ PFUs/mouse of SARS-CoV-2 (MAD6 isolate, containing D614G mutation) (**Figure 3A**). Challenged mice previously inoculated with one IN dose of MVA-WT were used as a control group.

To evaluate the vaccine efficacy, mice body weight and mortality were monitored during 14 days after the challenge. All K18-hACE2 mice immunized intranasally with one dose of MVA-Δ-S or MVA-S(3P) and challenged with SARS-CoV-2 did not lose body weight (**Figure 3B**) and survived (**Figure 3C**), whereas mice inoculated with MVA-WT and challenged with SARS-CoV-2 lost more than 20% of body weight (**Figure 3B**) and all were sacrificed at 8 days postchallenge (**Figure 3C**). Mice immunized with one IN dose of MVA-S lost body weight during the first 6 days postchallenge (**Figure 3B**), but recovered and survived (**Figure 3C**).

To determine the effect of IN vaccination in SARS-CoV-2 virus replication, four mice per group were sacrificed at day 5 after SARS-CoV-2 virus challenge, lungs and nasal washes were collected and processed, and the presence of SARS-CoV-2 subgenomic E and genomic RdRp mRNA (**Figures 3D, E**), as well as of live infectious virus (**Figures 3F, G**) was analyzed. MVA-S(3P) was more effective than MVA-S or MVA-Δ-S to prevent SARS-CoV-2 replication in the lungs (**Figure 3D**), but all vaccine candidates reduced significantly SARS-CoV-2 subgenomic and genomic mRNA levels in lungs and nasal washes in comparison to MVA-WT control infected mice (**Figures 3D, E**). Remarkably, no infectious virus was detected in lungs (**Figure 3F**) or nasal washes (**Figure 3G**) from all vaccinated mice, in comparison to MVA-WT control mice, indicating that sterilizing immunity was achieved in the upper and lower respiratory tracts, in terms of infectious virus.

### Intranasal administration of one single dose of MVA-based vaccine candidates reduced lung pathology and levels of pro-inflammatory cytokines

**Figure 4:**
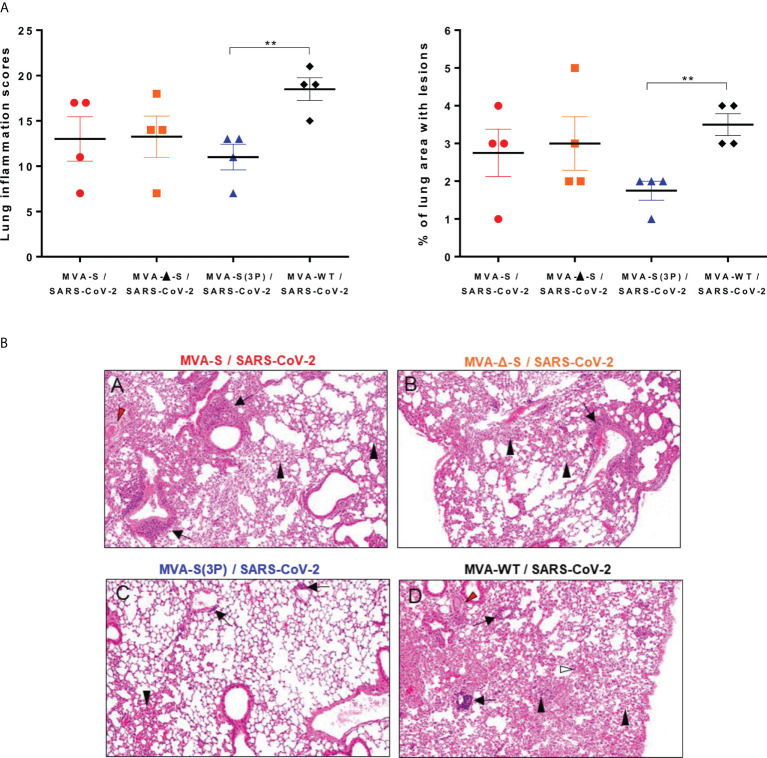
One IN dose of MVA-S(3P) reduced SARS-CoV-2 lung pathology in K18-hACE2 transgenic mice. **(A)** Lung inflammation scores (left) and percentage of lung area with lesions (right) examined in lung samples taken from mice (*n* = 4/group) vaccinated and infected as indicated in **Figure 3A**, and euthanized at 5 days postchallenge. Mean and SEM of cumulative histopathological lesion scores (left) and percentage of lung area affected by inflammatory lesions (right). Unpaired *t*-test: **p < 0.01. **(B)** Representative lung histopathological sections (H&E staining) from K18-hACE2 mice euthanized at day 5 postchallenge (magnification: 10×). Mice immunized with one dose of MVA-S **(A)** and MVA-Δ-S **(B)** displayed moderate inflammatory lung lesions that were, in general, more severe and extensive in mice immunized with MVA-WT (control infected group; d). These lesions highlighted the presence of diffuse thickening of the alveolar septae, perivascular edema (red arrowheads), mononuclear cell infiltrates within alveolar spaces (black arrowheads), large multifocal perivascular and peribronchiolar mononuclear infiltrates (black arrows), and occasional hemorrhages (white arrowheads). However, mice immunized with MVA-S(3P) **(C)** only displayed small lung areas with mild inflammatory lesions such as focal thickening of alveolar septae, occasional presence of mononuclear cell infiltrates within alveolar spaces (black arrowheads), and mild perivascular or peribronchiolar mononuclear infiltrates (black arrows).

Next, we evaluated the impact of IN vaccination on lung pathology and levels of pro-inflammatory cytokines in lungs and nasal washes. Histopathological evaluation of lungs at 5 days postchallenge (*n* = 4/group) showed that mice vaccinated intranasally with one dose of MVA-S(3P) displayed significant lower lung inflammation scores (**Figure 4A**, left panel) and lesser percentages of lung area with lesions (**Figure 4A**, right panel) than control MVA-WT mice. On the other hand, mice vaccinated with MVA-S or MVA-Δ-S showed slightly lower levels of lung inflammation scores and lesser percentages of lung area with lesions than control MVA-WT mice (**Figure 4A**), but differences were not significant. Representative images of lung sections clearly showed that mice vaccinated with one dose of MVA-S(3P) only displayed focal thickening of the alveolar septae, and occasional presence of inflammatory cells within the alveoli (**Figure 4B**). However, mice immunized with one dose of MVA-S or MVA-Δ-S or inoculated with control MVA-WT showed more severe diffuse thickening of the alveolar septae, higher presence of mononuclear cell infiltrates within alveolar spaces, and the presence of larger multifocal perivascular and peribronchiolar mononuclear infiltrates (**Figure 4B**).

**Figure 5:**
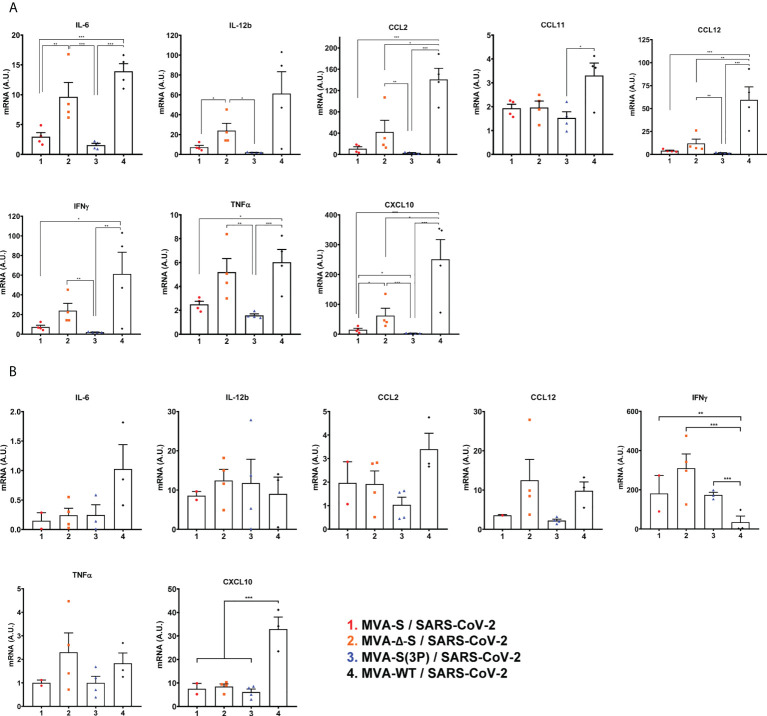
One IN dose of MVA-S(3P) diminished levels of pro-inflammatory cytokines in K18-hACE2 transgenic mice. mRNA levels of several cytokines/chemokines were detected by RT-qPCR in lungs **(A)** and nasal washes **(B)** obtained at 5 days postchallenge (*n* = 4/group). Mean RNA levels (in A.U.) and SEM from duplicates of each sample; relative values are referred to uninfected mice. Student’s *t*-test: *p < 0.05; **p < 0.005; ***p < 0.001.

Next, the effect of IN vaccination on the pro-inflammatory cytokine pattern induced in infected mice was evaluated at 5 days postchallenge by measuring by RT-qPCR the mRNA levels of key cytokines on the upper and lower respiratory tracts, lung homogenates (**Figure 5A**), and nasal washes (**Figure 5B**). Remarkably, compared to control MVA-WT infected mice, one IN dose of MVA-S(3P) induced a significant downregulation of several pro-inflammatory cytokines, such as IL-6, IL-12b, CCL2, CCL11, CCL12, IFN-γ, TNF-α, and CXCL10, in lung samples (**Figure 5A**). Similar results were obtained in nasal washes, except for IL-12b and IFN-γ (**Figure 5B**). Moreover, MVA-S- or MVA-Δ-S-vaccinated mice also induced downregulation in several cytokines, but the levels detected were higher than those elicited by MVA-S(3P)-vaccinated mice (**Figures 5A, B**).

### S-specific IgGs and neutralizing antibodies against different SARS-CoV-2 variants of concern in immunized K18-hACE2 transgenic mice before and after SARS-CoV-2 infection

Next, we assessed, in serum samples, SARS-CoV-2-specific humoral immune responses induced in transgenic K18-hACE2 mice vaccinated with one IN dose of MVA-S, MVA-Δ-S, or MVA-S(3P), after vaccine immunization or post-SARS-CoV-2 challenge.

**Figure 6:**
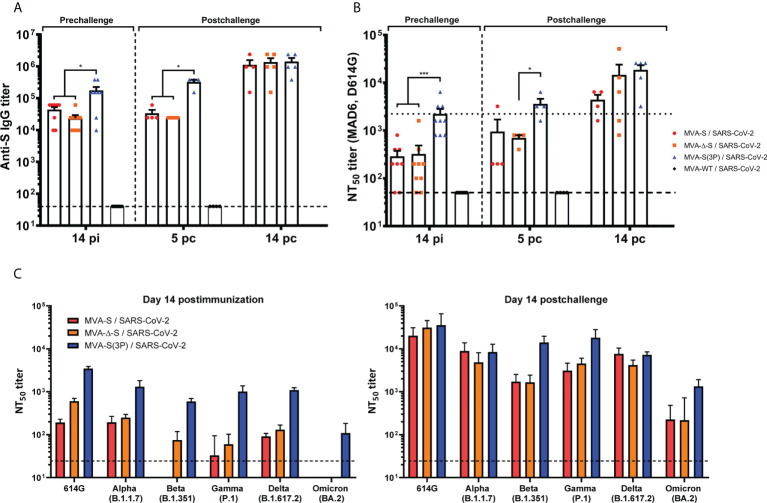
MVA-based vaccine candidates induced high levels of SARS-CoV-2-specific humoral immune responses in vaccinated and challenged K18-hACE2 transgenic mice. **(A)** Titers of IgG antibodies specific for the S protein (Wuhan strain). Determined by ELISA in individual mouse serum samples collected at day 14 postimmunization (pi) (prechallenge; *n* = 9/group) and at days 5 (*n* = 4/group) and 14 (*n* = 5/group) postchallenge (pc) from K18-hACE2 mice. Mean values and SEM are represented. The dashed line represents the limit of detection. **(B)** SARS-CoV-2 neutralizing antibody titers. NT_50_ titers were evaluated in individual mouse serum samples collected at day 14 pi and at days 5 and 14 pc, using a live virus microneutralization assay (MAD6 strain, having D614G mutation). Mean NT_50_ values and SEM are represented. The dotted line represents the limit of detection. **(C)** SARS-CoV-2 neutralizing antibody titers against SARS-CoV-2 VoC. NT_50_ titers were evaluated in pooled mouse serum samples collected at days 14 pi (left) and 14 pc (right), using VSV-based pseudoparticles expressing the SARS-CoV-2 S protein of different VoC. Mean NT_50_ values and 95% confidence intervals are represented. The dashed line represents the limit of detection. Student’s *t*-test: *p < 0.05; ***p < 0.001.

K18-hACE2 mice immunized with one IN dose of MVA-S(3P) induced, at 14 days postimmunization, significantly higher titers of anti-S total binding IgG antibodies than MVA-S- or MVA-Δ-S-vaccinated mice (**Figure 6A**). Similar differences were detected 5 days after SARS-CoV-2 infection; nonetheless, all vaccinated groups showed comparable IgG titers against S at day 14 postchallenge (**Figure 6A**), reflecting an expansion effect due to the infection in the MVA-S- and MVA-Δ-S-vaccinated groups, and the absence of a breakthrough infection in MVA-S(3P)-vaccinated mice, reflecting that MVA-S(3P) vaccination controlled the infection better.

Analysis of IgG isotypes against S protein at the prechallenge and postchallenge time points showed superior titers of IgG2c than IgG1 and lower titers of IgG3 antibodies in all immunization regimens, leading to a IgG2c/IgG1 ratio above 1 (**Table 1**), indicative of a Th1-like protective humoral response.

Consistently and similarly to anti-S IgG antibody titers, one IN dose of MVA-S(3P) induced significantly higher titers of neutralizing antibodies against live SARS-CoV-2 (MAD6 isolate) than MVA-S or MVA-Δ-S at 14 days postimmunization and at 5 days postchallenge, although at day 14 postchallenge, the neutralization titers increased and they were similar in all groups (**Figure 6B**), reflecting the existence of a breakthrough infection mainly in the MVA-S- and MVA-Δ-S-vaccinated groups.

Remarkably, the analysis of neutralizing antibodies against several VoC by using a VSV-pseudotyped neutralization assay showed that, at 14 days postimmunization, mice immunized with MVA-S(3P) induced neutralizing antibodies against all VoC tested, with NT_50_ neutralizing antibody titers markedly higher than the titers in MVA-S- or MVA-Δ-S-vaccinated mice (**Figure 6C**, left). NT_50_ neutralizing antibody titers against SARS-CoV-2 parental D614G mutant, VoC alpha (B.1.1.7), gamma (P.1), and delta (B.1.167.2) were similar, whereas the NT_50_ titers with VoC beta (B.1.351) were slightly lower and with the VoC omicron (BA.2; B.1.1.529.2) about 10 times lower (**Figure 6C**, left). SARS-CoV-2 infection markedly boosted neutralization titers against SARS-CoV-2 parental D614G mutant and all VoC at day 14 postchallenge in all vaccinated groups, with differences in NT_50_ titers similar to those described after vaccination (**Figure 6C**, right).

## Discussion

The high transmission of SARS-CoV-2 and its VoC among vaccinated individuals with one to three doses of the approved vaccines raise concern on the control of virus spreading among the population. As the majority of the clinical trials and vaccination programs have administered the vaccines by the intramuscular route (COVID-19 vaccine candidates already authorized are based on different approaches, such as inactivated, mRNA-, adenovirus-, and protein-based vaccines), and as it is well known that SARS-CoV-2 enters the respiratory tract to initiate virus infection with subsequent spread to the lungs and other tissues, further exploration of the mucosal route should be undertaken.

Immunization through intramuscular route elicits strong systemic immune responses, but it has a fundamental limitation as it is not effective in generating efficient mucosal immunity required to halt the SARS-CoV-2 infection of the upper respiratory mucosa, and prevent SARS-CoV-2 dissemination into the lower respiratory tract and subsequent pneumonia (46). Local mucosal immune responses such as airway epithelium T-cell responses and IgA humoral responses are critical for restricting respiratory viral pathogens, and these adaptive immune responses in the respiratory tract and the lung are required to achieve sterilizing immunity to virus re-infection (47–49). Thus, IN vaccination is an attractive route to elicit both antigen-specific mucosal and systemic immune responses able to induce sterilizing immunity (48). In the context of SARS-CoV-2/COVID-19, a recombinant adenovirus-based vaccine candidate has shown promising preclinical results when administered by the IN route (4, 7, 8) and has advanced to phase 1 clinical trials. Moreover, it has been recently reported in mouse and hamster animal models that IN vaccination with MVA-based vaccine candidates against SARS-CoV-2 is safe, immunogenic, and effective (14, 15). Furthermore, MVA-based vaccines against other infectious diseases, when delivered *via* the respiratory tract, were also proved to be well-tolerated and immunogenic in preclinical (50, 51) and clinical (52) studies.

Here, we demonstrated with three recombinant MVA-based vaccine candidates expressing either the native or a prefusion-stabilized SARS-CoV-2 S protein that a single IN administration of each vaccine was quite effective to induce robust immunogenicity and to better control SARS-CoV-2 infection in mice. The higher immunogenicity and efficacy of the MVA-S(3P) vaccine candidate, in comparison to MVA-S or MVA-Δ-S, agreed with our reported observations in mice immunized intramuscularly (26) and extended our previous investigations showing that MVA-based vaccines administered intramuscularly induced potent SARS-CoV-2-specific T-cell and humoral immune responses in several animal models, and fully protected against SARS-CoV-2 infection (16, 19, 20, 22, 25, 26). Interestingly, the IN administration of the MVA-based vaccine candidates elicited in mice systemic SARS-CoV-2-specific humoral and T-cell (spleen) immune responses as that seen with the intramuscular route (19, 20, 22, 26). Nonetheless, one single IN dose of the vaccine candidates MVA-S, MVA-Δ-S, and MVA-S(3P) elicited anti-S IgA antibodies in the mucosa, and they also induced local (in lungs and bronchial lymph nodes) S-specific CD4^+^ and CD8^+^ T-cell immune responses. It has been described that the limited SARS-CoV or SARS-CoV-2 viral replication in the lungs of intranasally vaccinated mice and hamsters correlated with the titers of antigen-specific IgA antibodies (4, 8, 53). In addition, mucosal IgA in saliva (54, 55) and nasopharynx (55) is fundamental to neutralize SARS-CoV-2 infection and prevent its spread. IgA antibodies are the most essential effector molecule in the mucosa (56) and, together with tissue-resident T cells directed against the S protein, are presumed, by previous studies, to promote a rapid clearance of SARS-CoV-2 with the minimal immune response (14, 15). Our results clearly agreed with these findings, as we observed complete reduction of viral loads in the lungs and nasal washes after SARS-CoV-2 infection of IN vaccinated K18-hACE2 transgenic mice, as well as a significant reduction in lung pathology and in the levels of pro-inflammatory cytokines. We detected a minor amplification of SARS-CoV-2 subgenomic E mRNA early after challenge in nasal washes and lungs of K18-hACE2 transgenic mice vaccinated with one IN dose of MVA-S(3P), indicating that presumably virus replication was not entirely prevented by one IN administration. However, the lack of live infectious virus in both nasal washes and lungs denote that the infection was rapidly cleared, and perhaps nearly sterilizing immunity was obtained. In addition, the minimal cell infiltration in the lungs, mild lung histopathological lesions, and the significant lower levels of pro-inflammatory cytokines in the lungs of SARS-CoV-2-challenged K18-hACE2 transgenic mice vaccinated with MVA-S(3P) indicated very low virus transmission to the lower respiratory tract. Moreover, lack of live virus in nasal washes pointed to a potent immune response in the upper respiratory tract, which could prevent lung damage by virus replication. This effect could be potentiated by new technologies and administration devices that allow vaccine retention in the nasal cavity and lymphoid associated tissues (57). Moreover, SARS-CoV-2-specific immune responses elicited by our MVA-based vaccine candidates in the lower respiratory tract also reflect that MVA-based vaccine candidates are capable of reaching the lungs, as it has been reported (14, 58). Nonetheless, all these data suggested that perhaps it is very difficult to eliminate completely the input virus by the immune system. Vaccination with two IN doses could further enhance the control of SARS-CoV-2 infection.

Remarkably, the efficacy elicited in mice after one IN immunization with our MVA-based vaccine candidates against COVID-19 was superior to the SARS-CoV-2 control of infection induced after one intramuscular administration (22, 26). In particular, SARS-CoV-2 subgenomic and genomic mRNA levels and titers of infectious virus in lungs of K18-hACE2 mice vaccinated with MVA-S or MVA-S(3P) were significantly reduced after one IN vaccination than after intramuscular inoculation, reflecting a better control of SARS-CoV-2 infection. In fact, no infectious virus was detected after one IN vaccination of all MVA-based vaccine candidates, while low levels were detected after one intramuscular inoculation (22, 26).

Our results, and those from other groups (14, 15), demonstrate the benefits of IN administration of MVA-based vaccine candidates against COVID-19, although there were some differences between our results and those reported by other groups. On the one hand, Bošnjak et al. observed strong cellular but weak humoral S-specific immune responses after one single IN application of their MVA-based vaccine candidates (15), whereas we observed high titers of S-specific IgG antibodies, as well as SARS-CoV-2 neutralizing antibodies, even against several VoC. On the other hand, Americo et al. observed that IN administration of two doses of their MVA-based SARS-CoV-2 vaccine candidates led to induction of more IgA and CD8^+^ T cells than intramuscular administration, but similar levels of IgG and neutralizing antibodies (14). Similarly, we observed similar titers of anti-S IgG and neutralizing antibodies in serum samples from mice immunized with one IN or intramuscular dose of our MVA-based vaccine candidates (19, 22, 26).

Although it has been recently described that two IN vaccinations of MVA-based SARS-CoV-2 vaccine candidates induce higher antibody titers with stronger neutralization potency and increase T-cell responses than one dose (14), we observed that one single IN dose of our MVA-based vaccine candidates is highly efficacious, and is sufficient to prevent morbidity and mortality in SARS-CoV-2-challenged K18-hACE2 transgenic mice, reducing SARS-CoV-2 virus replication in upper and lower respiratory tracts, lung pathology, and the levels of pro-inflammatory cytokines. Moreover, one IN dose of our MVA-based vaccine candidates triggered high titers of S-specific IgGs and neutralizing antibodies against different SARS-CoV-2 VoC in K18-hACE2 transgenic mice. These results are in accordance with previous reports, where very effective neutralizing antibodies were found against Wuhan, alpha, and delta SARS-CoV-2 VoC, while protection against beta and gamma variants was less prominent (14, 15). Nevertheless, the high levels of neutralizing antibodies elicited by MVA-S(3P) against VoC beta, delta, and even omicron suggest that this optimized MVA-based vaccine candidate could protect against different VoC.

Collectively, our data illustrated the advantages of IN immunization of our optimized MVA-S(3P) vaccine candidate and support the possibility of delivering MVA-vectored SARS-CoV-2 vaccines by the IN route to prevent the virus transmission among the human population. It would be extremely interesting to investigate in clinical trials the use of IN vaccination with MVA-based vaccines against SARS-CoV-2, as well as their potential as a booster dose in previously vaccinated individuals with different COVID-19 vaccines.

## Data availability statement

The original contributions presented in the study are included in the article/supplementary material. Further inquiries can be directed to the corresponding authors.

## Ethics statement

Female C57BL/6OlaHsd mice (6-8 weeks old) used for immunogenicity assays were purchased from Envigo Laboratories and stored in the animal facility of the Centro Nacional de Biotecnología (CNB) (Madrid, Spain). Female transgenic K18-hACE2 mice, expressing the human angiotensin converting enzyme-2 (ACE2) receptor, were obtained from the Jackson Laboratory [034860-B6.Cg-Tg(K18-ACE2)2Prlmn/J, genetic background C57BL/6J x SJL/J)F2], and efficacy experiments were performed in the biosafety level 3 (BSL-3) facilities at the Centro de Investigación en Sanidad Animal (CISA)-Instituto Nacional de Investigaciones Agrarias (INIA-CSIC) (Valdeolmos, Madrid, Spain). The immunogenicity and efficacy animal studies were approved by the Ethical Committee of Animal Experimentation (CEEA) of the CNB-CSIC and by the Division of Animal Protection of the Comunidad de Madrid (PROEX 49/20, 169.4/20 and 161.5/20). Animal procedures conformed with international guidelines and with Spanish law under the Royal Decree (RD 53/2013).

## Author contributions

Conceptualization: ME and JG-A. Formal analysis: PP, DA, GA, and JG-A. Funding acquisition: RD, JC, ME, and JG-A. Investigation: PP, DA, GA, SF, PS-C, JL, and JG-A. Methodology: PP, DA, GA, PS-C, JL, RD, and JG-A. Resources: JC and RD. Supervision: ME and JG-A. Validation: PP, DA, GA, JL, and JG-A. Visualization: PP, DA, GA, and JG-A. Writing—original draft: PP, ME, and JG-A. Writing—review and editing: all authors. All authors contributed to the article and approved the submitted version.

## Funding

This research was supported by Fondo COVID-19 grant COV20/00151 [Spanish Health Ministry, Instituto de Salud Carlos III (ISCIII)], Fondo Supera COVID-19 grant (Crue Universidades-Banco Santander), and Spanish Research Council (CSIC) grant 202120E079 (to JG-A); CSIC grant 2020E84, la Caixa Banking Foundation grant CF01-00008, Ferrovial, and MAPFRE donations (to ME); and Spanish Ministry of Science and Innovation (MCIN)/Spanish Research Agency (AEI)/10.13039/501100011033 grant (PID2020-114481RB-I00; to JG-A and ME). This research work was also funded by the European Commission-NextGenerationEU, through CSIC’s Global Health Platform (PTI Salud Global) (to JG-A and ME). JG-A and ME acknowledge financial support from the Spanish State Research Agency, AEI/10.13039/501100011033, through the “Severo Ochoa” Programme for Centres of Excellence in R&D (SEV-2013-0347, SEV-2017-0712). JMC acknowledges MCIN and CSIC support (project number 202020E079). RD received grants from ISCIII (FIS PI2100989), the European Commission Horizon 2020 Framework Programme (Project VIRUSCAN FETPROACT-2016: 731868 and Project EPIC-CROWN-2: 101046084), and Fundacioín Caixa-Health Research HR18-00469 (Project StopEbola).

## Acknowledgments

We thank CSIC and MCIN for continuous support. We thank Centro de Investigación en Sanidad Animal (CISA)-Instituto Nacional de Investigaciones Agrarias (INIA-CSIC) (Valdeolmos, Madrid, Spain) for the BSL-3 facilities. SARS-CoV-2 MAD6 virus isolate was kindly provided by José M. Honrubia and Dr. Luis Enjuanes (CNB-CSIC, Madrid, Spain). We thank the Histology Facility at CNB-CSIC for histological preparation of biological samples. We thank Carlos Óscar Sánchez Sorzano from the CNB Bioinfomatic Unit for help with the statistical analysis. We thank Iván Jareño from the CNB Animal Facilities for help and assistance.

## Conflict of interest

The authors declare that the research was conducted in the absence of any commercial or financial relationships that could be construed as a potential conflict of interest.

## Publisher’s note

All claims expressed in this article are solely those of the authors and do not necessarily represent those of their affiliated organizations, or those of the publisher, the editors and the reviewers. Any product that may be evaluated in this article, or claim that may be made by its manufacturer, is not guaranteed or endorsed by the publisher.
